# To Protect and to Preserve: Novel Preservation Strategies for Extracellular Vesicles

**DOI:** 10.3389/fphar.2018.01199

**Published:** 2018-10-29

**Authors:** Gina D. Kusuma, Mehri Barabadi, Jean L. Tan, David A. V. Morton, Jessica E. Frith, Rebecca Lim

**Affiliations:** ^1^The Ritchie Centre, Hudson Institute of Medical Research, Clayton, VIC, Australia; ^2^Department of Obstetrics and Gynaecology, Monash University, Clayton, VIC, Australia; ^3^Department of Materials Science and Engineering, Monash University, Clayton, VIC, Australia; ^4^Australian Regenerative Medicine Institute, Monash University, Clayton, VIC, Australia; ^5^School of Engineering, Deakin University, Geelong, VIC, Australia

**Keywords:** nanomedicine, exosomes, extracellular vesicles, biomaterials, cryopreservation, regenerative medicine, biologics

## Abstract

Extracellular vesicles (EVs)-based therapeutics are based on the premise that EVs shed by stem cells exert similar therapeutic effects and these have been proposed as an alternative to cell therapies. EV-mediated delivery is an effective and efficient system of cell-to-cell communication which can confer therapeutic benefits to their target cells. EVs have been shown to promote tissue repair and regeneration in various animal models such as, wound healing, cardiac ischemia, diabetes, lung fibrosis, kidney injury, and many others. Given the unique attributes of EVs, considerable thought must be given to the preservation, formulation and cold chain strategies in order to effectively translate exciting preclinical observations to clinical and commercial success. This review summarizes current understanding around EV preservation, challenges in maintaining EV quality, and also bioengineering advances aimed at enhancing the long-term stability of EVs.

## Introduction

Interest in extracellular vesicles (EVs) has escalated over the last decade. This has been particularly the case in clinical applications, including the application of EV biology to biomarker discovery, vaccine development, drug delivery, and EV-based therapeutics. EVs are key players in intercellular communication and they are protected from degradation by their lipid bilayer membrane that envelop bioactive cargo. These include proteins, sugars, lipids, and nucleic acids. EVs can be classified based on their size, i.e., apoptotic bodies (>1000 nm), microvesicles (100 – 1000 nm), and exosomes (30 – 150 nm) (Kalra et al., 2016; Tkach and Théry, 2016). The field of EV research is rapidly gaining momentum and overlaps with the newer field of bioengineering where synthetic liposomes, biomimetic vesicles, and nanoparticles have been utilized to package bioactive cargo. In this review, we assess current strategies employed for EV preservation and bioengineering advances aimed at enhancing long term stability of EVs intended for clinical use.

### Composition and Cargo of EVs

Extracellular vesicles are ideal intercellular transporters of biomolecules. They express surface molecules that enable tissue- or cell-specific targeting. Upon reaching their recipient cells, EVs can induce signaling via receptor-ligand interaction, or be internalized by endocytosis to deliver their cargo. The term “exosomes” is generally used to describe most EVs globally. In an attempt to standardize nomenclature and improve accuracy of data interpretation, the International Society of Extracellular Vesicles (ISEV) published a set of guidelines in 2014 that outlined the so-called minimal requirements to define EVs (Lötvall et al., 2014). The collective term of EVs will be used throughout this review since the definition of exosomes remains contentious. Given the increasing interest in EVs and their potential use in regenerative medicine, isolated EVs must be carefully characterized – this necessarily requires a complex combination of protein profiling (proteomics, western blotting, or flow cytometry), imaging, and nanoparticle tracking analysis.

Extracellular vesicles are secreted by virtually all cell types and present in all bodily fluids. An online public database, ExoCarta^1^ (Keerthikumar et al., 2016) has been created to curate this diverse body of data, with the goal of facilitating and encouraging collaborative research. This public repository is being continuously updated with new contributions from various EV researchers.

Extracellular vesicles are enriched in membrane proteins and cellular proteins, including the tetraspanins CD63, CD9, CD81, Alix, Tsg101, MHC1 and heat shock proteins (van Niel et al., 2006; Raposo and Stoorvogel, 2013). The protein cargo of EVs include cell-specific proteins which are responsible for specific fates and functions, such as: cell adhesion (integrins, ICAM), signal transductions (G proteins, β-catenin, protein kinases), and intracellular trafficking (RAB, GTPases, annexins) (van Niel et al., 2018). The lipid contents of EVs include ceramides, sphingomyelins, phosphatidylserine, and cholesterol (Laulagnier et al., 2004; Subra et al., 2007). This unique lipid composition is thought to facilitate the uptake of EVs by recipient cells. The lipid composition of EV membranes also play significant roles in intercellular signaling and provide structural stability (Skotland et al., 2017). Furthermore, the surfaces of EVs are surrounded by polysaccharides and glycan (Batista et al., 2011). The nucleic acid cargo in EVs such as mitochondrial DNA, genomic DNA, mRNA, miRNA, and long non-coding RNA have already been documented extensively. Importantly, exosomal RNA play functional roles in EV-mediated cellular communication where exosomal mRNA can be translated into proteins in recipient cells and exosomal microRNA (miR) may regulate gene expression in recipient cells (Valadi et al., 2007).

### EVs Molecular Cargo Involved in Therapeutic Benefits/Immunomodulation

EV-mediated delivery is an effective and efficient system to confer therapeutic benefits to their target destinations. The contents of EV cargo can be heavily influenced by their producer cells and different cell types will secrete a range of functional effects on recipient cells. The ability of EVs to interact with recipient cells is likely to be affected by the presence of adhesion molecules (e.g., integrins) on the surface of EVs, and this will contribute to the cell or tissue specificity of EVs (van Niel et al., 2018).

Extracellular vesicles exhibit intrinsic therapeutic benefits, for example, EVs can be used as gene delivery vehicles without inducing adverse immune reactions. This contrasts with the more commonly used gene therapy vehicles such as viral vectors and lipid nanoparticles which are immunogenic (Kumar et al., 2014).

There are a number of different strategies to identify and validate EV-mediated cargo delivery into recipient cells. For example, labeling EVs with a tracking dye can result in a quantifiable increase in fluorescence in the recipient cells once exosome uptake occurs. Alternatively, EV-associated RNA labeled with a radioactive tracer can be used to demonstrate uptake by recipient cells (Valadi et al., 2007). For the purposes of this review, we have summarized recent studies describing the therapeutic use of EVs from human cell types in Table 1.

**Table 1:** Individual human-derived EVs cargo components and their therapeutic effects.

EV cargo	EV source	Recipient	Therapeutic claim	Reference
*Proteins*				
Peptide-MHC complexes	Dendritic cells pulsed with diphtheria toxin	Mice	Induced diphtheria-toxin antibody production	Colino and Snapper, 2006
APOBEC3G (antiviral protein)	Human CD4^+^ T cells	Jurkat T cells	Resistance to HIV	Khatua et al., 2009
Fas	hBMMSCs	Fas-deficient mice	Ameliorated osteopenia	Liu et al., 2015
EMMPRIN	CMPCs and MSCs	HMECs and HUVECs	Increased angiogenesis and endothelial cell migration	Vrijsen et al., 2016
AT1R	HEK293T cells	Mice	Modulated blood pressure	Pironti et al., 2015
Dll4	U87GM and HUVECs	HUVECs	Increased Notch signaling and angiogenesis	Sheldon et al., 2010
MHC class I and II	B cells	T cells	Induced T cell proliferation and T_H_2-like cytokine production	Admyre et al., 2007
Cystinosin (and *CTNS* mRNA)	hAMMSCs and hBMMSCs	Cystinotic fibroblasts	Reduced cystine accumulation	Iglesias et al., 2012
Neprilysin	hADMSCs	Mouse neuroblastoma cells	Decreased intracellular β–amyloid peptide	Katsuda et al., 2013
CD73	hBMMSCs	GVHD mice	Promoted adenosine-based immunosuppression	Amarnath et al., 2015
*Nucleic acids*				
mtDNA	hBMMSCs	Macrophages	Reduced mitochondrial ROS generation	Phinney et al., 2015
lncRNA	Hela cells	C33A cells	Enhanced cell viability	Hewson et al., 2016
mRNA (*Wnt4*)	UC-MSCs	Mice	Accelerated wound re-epithelisation and cell proliferation	Zhang et al., 2015a
mRNA (*IL-10*)	hBMMSCs and UC-MSCs	Kidney tubular cells	Increased cell recovery following injury	Ragni et al., 2016
mRNA (*IGF-1R*)	hBMMSCs	Cisplatin-damaged PTECs	Enhanced cell proliferation	Tomasoni et al., 2013
miR-150	Monocytes	Endothelial cells	Promote angiogenesis	Li et al., 2013
miR-143, miR-145	Endothelial cells	Aortic SMCs	Reduced atherosclerotic lesions	Hergenreider et al., 2012
Let-7c	hMSCs	Mice	Reduced renal fibrosis	Wang et al., 2016
miR-21, miR-210	iPSCs	Cardiomyocytes	Rescued ischemic cardiomyocytes	Wang et al., 2015
miR-146a	hMSCs	Macrophages	M2 polarization and increased survival in septic mice	Song et al., 2017
miR-21-3p	UCB plasma	Mice	Enhanced angiogenesis and promoted wound healing	Hu et al., 2018
miR-22	hMSCs	Cardiomyocytes	Improved cardiac function	Feng et al., 2014
miR-1343	HL-60 neutrophil-like cells	Lung fibroblasts	Inhibition of TGF-β signaling and myofibroblast differentiation	Stolzenburg and Harris, 2017
miR-100	hMSCs	Breast cancer cells	Suppression of angiogenesis and downregulation of VEGF	Pakravan et al., 2017
miR-19a	hMSCs	Cardiomyocytes	Restored cardiac contractile function and reduced infarct size	Yu et al., 2015
miR-21-5p	hMSCs	iPSCs-derived cardiomyocytes and iPSCs-derived fibroblasts	Increased engineered cardiac tissue contractility via PI3K signaling	Mayourian et al., 2018
miR-126, miR-296	EPCs	Islet endothelium	Increased angiogenesis and revascularisation of islets	Cantaluppi et al., 2012
miR-146a	CDCs	Injured mouse hearts	Inhibited apoptosis, promote cardiomyocytes proliferation and angiogenesis	Ibrahim et al., 2014
miR-196a	hBMMSCs	Rats with calvarial bone defects	Stimulated bone formation	Qin et al., 2016
miR-23b	hBMMSCs	Human breast cancer cell line	Induced dormant phenotype	Ono et al., 2014
miR-125a	hADMSCs	HUVECs	Promoted angiogenesis	Liang et al., 2016
miR-122	hADMSCs	Hepatocellular carcinoma cells	Increased sensitivity to chemotherapeutic agents	Lou et al., 2015


*EMMPRIN, extracellular matrix metalloproteinase inducer; CMPCs, cardiomyocyte progenitor cells; HMECs, human microvascular endothelial cells; HUVECs, human umbilical vein endothelial cells; AT1R, angiotensin II type I receptor; Dll4, Delta-like 4 Notch ligand; hAMMSCs, human amniotic mesenchymal stem cells; hBMMSCs, human bone marrow MSCs; hADMSCs, human adipose tissue MSCs; mtDNA, mitochondrial DNA; UC-MSCs, umbilical cord MSCs; PTECs, proximal tubular epithelial cells; SMCs, smooth muscle cells; iPSCs, induced pluripotent stem cells; UCB, umbilical cord blood; EPCs, endothelial progenitor cells; CDCs, cardiosphere-derived cells.*

EV-based therapeutics have been proposed as an alternative to cellular therapy, where the latter refers to the use of intact, living cells. In particular, cell therapy exploits the ability of the cellular products to secrete a complex repertoire of bioactive factors including EVs. However, the widespread use of cell therapies has been limited by challenges in the scalability and reproducibility of cell manufacturing. A paradigm shift toward cell-free therapies has now captured the attention of this sector, where the potential of EVs is being explored (Gnecchi et al., 2016; Kusuma et al., 2017). In comparison to cells, EVs have a simplified cold chain process, and have a lower risk profile due to the unlikelihood of spontaneous DNA transformation or immune rejection. Furthermore, EVs can be used directly, either alone or in combination with other pharmacological agents (Fais et al., 2016).

## EV-Based Therapeutics

### Preclinical Evaluation of EV-Based Therapeutics

Stem cell-derived EVs have been shown to modulate the immune response from both the innate and adaptive immunity. Favaro et al. (2014, 2016) showed that BMMSCs-EVs induced regulatory dendritic cell (DC) phenotypes with the ability to inhibit T cell activity, while ESC-EVs can reportedly promote M2 macrophage polarization, upregulate Treg numbers and downregulate splenocyte proliferation (Zhang et al., 2014a). Additionally, MSC-EVs were reported to promote Treg proliferation and inhibit autoreactive T cell activity (Del Fattore et al., 2015), as well as induce polymyxin-resistant MyD88-dependent secreted embryonic alkaline phosphatase expression in THP-1 cells (Zhang et al., 2014b). In a mouse model of myasthenia gravis, MSC-EVs reduced T cell-dependent immunoactivation, ameliorated autoimmune injury, and prolonged survival time (Sudres et al., 2017). Additionally, Shigemoto-Kuroda et al. (2017) showed that MSC-EVs modulate immune responses in two different autoimmune mouse models. In a mouse model of type I diabetes, they showed that MSC-EVs delayed the onset of type I diabetes through modulation of IL-1β mediated pancreatic B-cell destruction. Similarly, they showed that 30 μg of MSC-EVs attenuated uveoretinitis triggered by Th1/Th17 activation (Shigemoto-Kuroda et al., 2017).

In murine models of kidney injury, MSC-derived EVs protected against renal injury by reducing levels of creatinine, uric acid, lymphocyte response and fibrosis through shuttling miR-let7c to induce renal tubular cell proliferation (Wang et al., 2016). In a murine model of carbon tetrachloride-induced hepatic injury, concurrent treatments of MSC-EVs attenuated the injury by increasing the proliferation, survival and prevented the apoptosis of hepatocytes (Tan et al., 2014). In animal models of lung injury, MSC and hAEC-EVs have been shown to reduce pulmonary inflammation, improved lung tissue recovery and supported the proliferation of alveolar type II and bronchioalveolar stem cells (Rubenfeld et al., 2005; Cruz et al., 2015; Monsel et al., 2015; Tan et al., 2018). In models of stroke, MSC-EVs delivery of miR-133b directly to neurite cells reportedly enhanced the outgrowth of neurites resulting in increased proliferation of neuroblasts and endothelial cells (Xin et al., 2013). Additionally, Anderson et al. showed through a comprehensive proteomic analysis that MSC-derived EVs mediated angiogenesis via NF-κB signaling (Anderson et al., 2016), while Zhang et al. (2015b) showed that UC MSC-EVs mediated angiogenesis via the Wnt4/β-catenin pathway.

The possibility for EV-based therapeutics to be developed from immune cells is also currently being explored. EVs from dendritic cells have been engineered in various ways to help combat autoimmune diseases. These include stimulating DCs with IFNγ to express miRNAs which stimulate myelination, and reduce oxidative stress (Pusic et al., 2014). Immature DCs (iDCs)-EVs, which have not conformed to their mature role in expressing MHC and co-stimulatory molecules, displayed immunosuppressive properties in autoimmune diseases. For instance, in a mouse model of autoimmune neuromuscular disorder; myasthenia gravis (MG) iDC-derived EVs prevented MG disorder by suppressing lymphocyte reactivity *in vivo* (Bu et al., 2015). Immune cell-derived EVs are relatively easy to isolate and as such can be beneficial as potential targets for autoimmune and cancer treatments.

### Clinical Application of EV-Based Therapeutics

There is currently only a handful of clinical trials based on therapeutic EVs registered; all of which are currently still recruiting (Fais et al., 2016; Lener et al., 2015). However only one official trial has been reported to date using ascites-derived exosomes for the treatment of colorectal cancer (Dai et al., 2008). Additionally, in a letter to the editor, the use of stem cell-derived EV administered under compassionate care to patients suffering from graft vs. host disease (GvHD) recorded no adverse effects (Kordelas et al., 2014). The first study was dated back to 2008 (Dai et al., 2008), while the second was published in 2014 (Kordelas et al., 2014). Since then, there is a modest increase in the number of clinical trials with five out of seven using biologically derived EVs while the remaining are plant based EVs. These trials are currently recruiting and are expected to commence in the near future.

**FIGURE 1:**
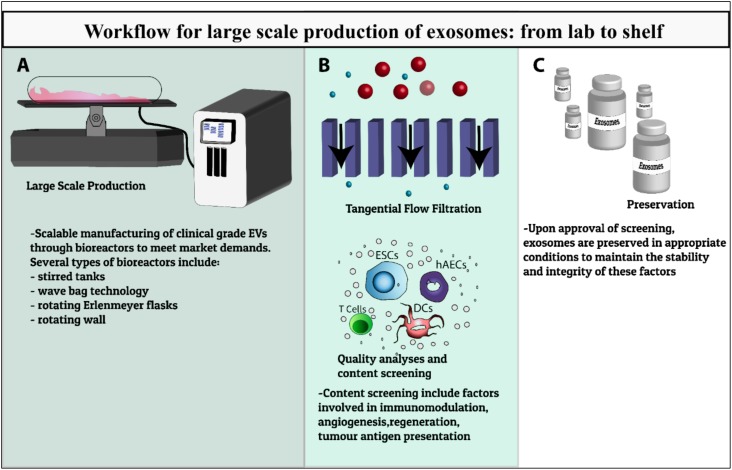
Workflow summary of EVs production for clinical use. Schematic of the development of EV therapeutics from preclinical testing to scalable bioprocesses including **(A)** development of large scale manufacturing of clinical grade EVs through various types of bioreactors, **(B)** characterization, quality analysis and content screening including factors involved in immunomodulation, angiogenesis, regeneration, tumor antigen presentation, **(C)** preservation in appropriate storage conditions to maintain the stability and integrity of these factors to meet clinical-scale demands.

Current methods for EV manufacturing are inadequate. Indeed, scalable manufacturing of clinical grade EVs to meet market demands will be a major challenge for this emerging sector for the foreseeable future (Figure 1). Given the unique attributes of EVs, considerable thought must be given to the preservation, formulation, and cold chain strategies in order to effectively translate exciting preclinical observations to clinical and commercial success.

## Current Preservation Strategies for EVs

### Conventional Methods for EVs Preservation

Since the commercial and clinical applications of EVs require standard criteria for long-term storage, cryopreservation methods have become a subject of growing interest. This section will describe the current understanding around EV preservation, challenges in maintaining EV stability, and their impact on long term storage and cold chain processes. Table 2 highlights the current preservation methods used in EV for therapeutics purposes.

**Table 2:** Current storage and preservation methods for EVs.

Preservation method	Storage temperature	Storage solution	EV source	Isolation method	Reference
Conventional Freezing	-80°C	PBS	BMMSCs	Ultracentrifugation	Vallabhaneni et al., 2015
	-80° C	PBS	hAECs	Ultracentrifugation	Zhao et al., 2017
				Ultrafiltration	
	-80°C	PBS	iMSCs	Ultracentrifugation	Hu et al., 2015
				Sucrose gradient	
				Ultrafiltration	
	-80°C	PBS	MSCs	Ultracentrifugation	Zhu et al., 2014; Pachler et al., 2017
	-80°C	PBS	Cardiac fibroblasts and iPSCs	PEG precipitation	Hu et al., 2016
	4°C, -80°C	PBS	MSCs	Ultracentrifugation	Xin et al., 2012
	-80°C	PBS	imDCs	Ultracentrifugation	Tian et al., 2014
				Ultrafiltration	
	-80°C	PBS	Mouse BMDCs	Ultrafiltration/diafiltration	Viaud et al., 2009
	-80°C	PBS	Mouse BMDCs	Ultracentrifugation	Damo et al., 2015
				Ultrafiltration	
	-80°C	PBS	BMDCs	Ultracentrifugation	Naslund et al., 2013
	-80°C	0.9% normal saline	Dendritic cells	Ultracentrifugation on a D_2_O/sucrose cushion	Morse et al., 2005
	-80°C	0.9% NACl	MSCs	PEG precipitation	Ophelders et al., 2016
	-20°C	PBS	Brain endothelial cells	Invitrogen^®^ Total Exosome RNA and Protein Isolation Kit	Yang et al., 2015
	-80°C	Total Exosome Isolation reagent	EPCs	Ultracentrifugation using Total Exosome Isolation reagent (GENESEED, China)	Ke et al., 2017
	-80°C	Serum-free medium 199 + 25 mM HEPES	ADMSCs	Ultracentrifugation	Eirin et al., 2017
	-80°C	Serum-free medium 199 + 25 mM HEPES	HUVECs	Ultracentrifugation	Zhang et al., 2014c
	-80°C	RPMI + 1% DMSO	HK-2	Ultracentrifugation	Lindoso et al., 2014
	+4°C, -80°C	PBS + 25 mM Trehalose	MIN6 cells	Ultracentrifugation	Bosch et al., 2016
	-80°C	Serum-free Medium 199	MSC	Ultracentrifugation	Bruno et al., 2009, 2012


			Fibroblasts		
	-80°C	Medium 199	EPCs	Ultracentrifugation	Deregibus et al., 2012
			Fibroblasts		
	-80°C	Not disclosed	ESC-derived MSCs	Chromatography	Arslan et al., 2013
				Ultrafiltration	
	-80°C	Not disclosed	EPCs	Ultracentrifugation	Li et al., 2016
				Filtration	
	+4°C, +37°C, -20° C	Not disclosed	HEK293T, ECFC, MSCs	Ultracentrifugation	Sokolova et al., 2011
	+60°C, +37°C, +4°C, -20°C, -80°C	Not disclosed	HEK293T	ExtraPEG reagent	Cheng et al., 2018
Freeze drying	+4°C, -20°C, -80°C	Plasmalyte A, Ringers, Plasmalyte A + Dextrose	Cardiosphere-derived cells	Ultrafiltration	Kreke et al., 2016
				Diafiltration	
	-20°C	Laemmli Buffer	TM cells	Ultracentrifugation	Stamer et al., 2011
	-80°C	PBS	LIM1215 cells	Ultracentrifugation	Lydic et al., 2015


*BMMSC, human bone marrow mesenchymal stem cells; hAECs, human amniotic epithelial cells; iMSCs, iPSCs, imDCs, BMDCs, ADMSCs: adipose tissue MSCs; HUVECs, human umbilical vein endothelial cells; HK-2, human kidney cell line; MIN6, murine pancreatic beta cell line; ESC-derived MSCs, human embryonic stem cell-derived MSCs; HEK293T, human embryonic kidney cells; ECFC, endothelial colony forming cells; TM, human trabecular mesh cells; LIM1215, human colorectal cancer cell line.*

#### Cryopreservation

Cryopreservation with cryoprotectants (CPAs) is a widely accepted procedure to maintain protein stability and prevent osmotic damage (Elliott et al., 2017). Optimum EV dehydration can be achieved in the presence of CPAs by increasing viscosity, impacting the kinetics of ice nucleation, and allowing regulated extracellular ice growth during controlled cooling. However, excessively low concentrations of CPAs may result in chilling shock, which is defined as the damage caused by the freezing process. On the other hand, excessively high concentrations of CPAs can be toxic. Thus, a balance is needed to achieve optimal cryopreservation (Best, 2015).

**FIGURE 2:**
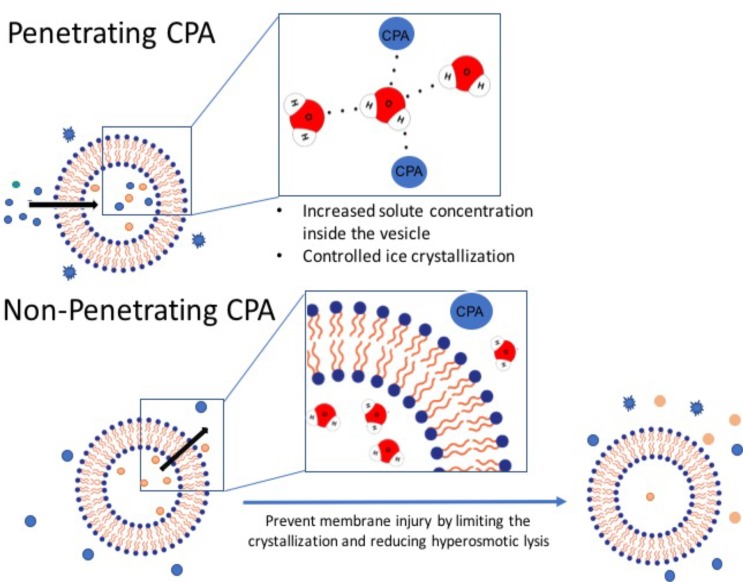
Penetrating vs. non-penetrating CPAs. Penetrating CPAs are low molecular weight molecules that can cross the lipid bilayer membrane and typically must be soluble in water, non-toxic, and can remain in solution at very low temperature. Non-penetrating CPAs have higher molecular weight; and by definition they do not permeate through the membrane and generally utilized at lower concentrations.

CPAs refer to a diverse range of sugars, diols, and amino acids which work to stabilize biomolecules in a variety of ways depending on their molecular mass, examples of CPA application in molecular and cell biology is described on Table 3. Penetrating CPAs (pCPA) have low molecular weights (<100 Da) and work by permeating across the lipid bilayer membranes to stabilize the biomolecules (Figure 2). In contrast, non-penetrating CPAs (npCPA) remain external to the vesicle due to their high molecular mass (180–594 Da) and prevent cryodamage from hyperosmotic lysis (Jan et al., 2008; Motta et al., 2014). Notably, there is a growing body of evidence suggesting a combination of both pCPAs and npCPAs is more effective (Table 3) (Willison and Rowe, 1980; Ha et al., 2005).

**Table 3:** Cryoprotective agents (CPA) used in cryopreservation of biological materials.

	Penetrating CPA	Non-penetrating CPA	Cocktails	Commercially available CPA
Nanoparticles	Glycerol(Sameti et al., 2003)	Trehalose, sucrose, fructose, glucose, sorbitol (10%) (Fonte et al., 2012)	20% Trehalose/Fructose (Date et al., 2010)	
	Gelatine(Schwarz and Mehnert, 1997)	Mannitol (Alihosseini et al., 2015)	Trehalose/Sucrose (Almalik et al., 2017)	
	Hydroxypropyl-β-cyclodextrin (Abdelwahed et al., 2006a,b)	Trehalose (Subedi et al., 2009)	10% DMSO/0.2 M sucrose (Marquez-Curtis et al., 2015)	
	Polyvinyl alcohol (Quintanar-Guerrero et al., 1998; Abdelwahed et al., 2006a)	Mannitol-dextrose-sucrose in ratio of 1:3, 1:2, and 1:1 (Patel et al., 2011)		
		Glucose (Quintanar-Guerrero et al., 1998; Kesenci et al., 2001; Abdelwahed et al., 2006a)		
		Lactose (Cui et al., 2003; (Hu et al., 2018^)^		
		Fructose (Zimmermann et al., 2000)		
		Dextran (Roy et al., 1997; Chacón et al., 1999)		
		Sucrose (Quintanar-Guerrero et al., 1998; Kesenci et al., 2001; Abdelwahed et al., 2006a)		
		Sorbitol (Storm et al., 1995; Kesenci et al., 2001; Panyam and Labhasetwar, 2012) Aerosil (colloidal silicon dioxide) (Schaffazick et al., 2003)		
Liposomes		Sucrose (Gala et al., 2015)		
		Trehalose (Harrigan et al., 1990; Hau et al., 2003; Nounou and El-Khordagui, 2005; El-Nesr et al., 2010; Nidhi et al., 2011)		
		Glucose, lactose, trehalose, and mannitol (Stark et al., 2010)		
Mammalian cells	DMSO (Bruder et al., 1997; Bozzo, 1999; Rust et al., 2006; Hendriks et al., 2010; Martinello et al., 2010; Thirumala et al., 2010; Chase et al., 2011; Xu et al., 2012; Dariolli et al., 2013; Chang et al., 2015)Ectoin (Heinrich et al., 2007; Sun et al., 2012; Bissoyi and Pramanik, 2013)Hydroxyectoin (Sun et al., 2012) 0.5, 1, or 1.5 M EG or propylene glycol or DMSO (Woods et al., 2010)	Trehalose (Beattie et al., 1997; Eroglu et al., 2000; Ann, 2005; Katenz et al., 2007; Motta et al., 2014; Tanaka et al., 2014; Rao et al., 2015; Cardoso et al., 2017; Martinetti et al., 2017)	DMSO + Trehalose (Chen et al., 2016) 2% DMSO in DMEM (Thirumala et al., 2010) Proline (1%) + ectoin (10%) (Freimark et al., 2011) Ectoine + trehalose + PEG (El Assal et al., 2014)PVP (Damjanovic and Thomas, 1974; Ray et al., 2016; Wiki) DMSO + 0.2 M sucrose (Roy et al., 2014)1,2-propanediol (Huang et al., 2015)Sucrose (Carrasco-Ramírez et al., 2016)0–10% DMSO + 0-10% HES (Naaldijk et al., 2012) DMSO or glycerol (5 or 10%) + sucrose (30 or 60 mM) + Trehalose (60 or 100 mM) (De Lara Janz et al., 2012)10% DMSO or 10% glycerol or 10% ethylene glycol (Ding et al., 2010)	Cellbanker (commercial-DMSO based) (Kotobuki et al., 2005; Edamura et al., 2014; Nam et al., 2014)50% Cryoprotective Medium (Lonza, Allendale, NJ, United States), 25% RPMI-1640, and 25% FBS (Jong et al., 2017)
Embryos and oocytes	PG/DMSO/EG (Trad et al., 1999)	PVP (Kim et al., 2008)		
		Trehalose (Eroglu et al., 2002)		
Proteins	Proline (Pemberton et al., 2012)	Sucrose (Crowe et al., 1987)		
		Trehalose (Jain and Roy, 2009; Lee, 2014)		
Tissues	DMSO (Casado-Díaz et al., 2008; Woods et al., 2010; Shen et al., 2012; Badowski et al., 2014; Choudhery et al., 2014; Lindemann et al., 2014)		40% EG/18% ficoll/0.3 M sucrose/20% FBS (Moon et al., 2008)	
			10% DMSO/10% EG/0.5 M sucrose (Dulugiac et al., 2015)	
			5% EG/35% PG/6% sucrose	
			5% EG/35% PG/5% sucrose/1% PVA (Wang et al., 2011)	
			40% EG/18% ficoll/0.3M sucrose/20% FBS (Kaviani et al., 2014; Shivakumar et al., 2015)	
			10% DMSO/5% Glycerol/0.2,0.5 M sucrose (Roy et al., 2014)	
			10% DMSO/5% Glycerol (Chatzistamatiou et al., 2014)	
EVs	DMSO (Wu et al., 2015b)	Trehalose (Bosch et al., 2016)		
	Albumin (Lörincz et al., 2014)			


*PG, propylene glycol; DMSO, dimethyl sulphoxide; EG, ethylene glycol; PVP, Polyvinyl pyrrolidine.*

A wide range of substances have been used as stabilizers in conventional cryopreservation methods. Specifically, disaccharides are a safe choice for EV-based therapeutics. Trehalose, a natural non-reducing disaccharide, is an FDA-approved CPA for a wide range of proteins and cell products (Eroglu et al., 2000; Buchanan et al., 2004; Motta et al., 2014; Bosch et al., 2016). Following reports showing the importance of adding pCPAs and npCPAs, trehalose was suggested as an ideal candidate to preserve hematopoietic and embryonic stem cells as well as other progenitor cells for therapeutic applications (Buchanan et al., 2004, 2005). Trehalose prevented aggregation by avoiding internal ice formation in biological particles such as liposomes and EVs (Bosch et al., 2016). The addition of trehalose also increased the colloidal stability of EVs (Hood et al., 2014).

#### Lyophilisation/Freeze Drying

Freeze-drying or lyophilisation is currently thought to be the most reliable method to preserve thermolabile materials such as proteins, peptides, vaccines, colloidal carriers, EVs and viruses (Khairnar et al., 2013; Hansen et al., 2015). The first step in lyophilisation involves the freezing or solidification of the EVs, when cooling rate correlates inversely with the size of the ice crystal. The crystallized material is then sublimated directly into water vapor. Freezing and dehydration stresses generated during lyophilisation may result in destructive effects on the structure of biomolecules within the EV, and thus necessitates the use of CPAs in the formulation to protect the EVs and their cargo (Wu et al., 2015a).

The stability of lyophilised EVs significantly extends their shelf life, lowers storage demands, and costs owing to a simplified cold chain. For example, the best storage temperature reported for lyophilised EVs isolated from cardiospheres was 4°C (Kreke et al., 2016). The most common stabilizers used in lyophilisation are disaccharides such as glucose, lactose, sucrose and trehalose, which work by replacing the hydration sphere around the EVs through a hydrogen bonding interaction with phospholipid head groups to form an amorphous sugar glass. The glassy state produced in the presence of disaccharides prevent fusion of products or protein destabilization (Jain and Roy, 2009).

Trehalose has been suggested as the most effective disaccharide to preserve EVs during lyophilisation (Chen et al., 2010; Bosch et al., 2016). This promising technique is an FDA-approved method for a range of proteins, liposomes and nanoparticles that enables their use in the pharmaceutical industry (Van Backstal et al., 2017).

#### Spray Drying

Spray drying is a common method for producing a wide variety of therapeutic agents including vaccines, peptides and proteins for inhaled delivery (Broadhead et al., 1992; Chan et al., 1997; Salama et al., 2009). This single-step process substantially reduces the need for expensive equipment and lengthier multi-step processes. Spray drying is scalable and operators are able to tune the particle size of the final product by controlling the spray droplet size and solute concentration, thereby providing a major point of difference from lyophilisation where the particle size reduction can occur only through mechanical milling (Costantino et al., 2000).

Spray drying involves an initial step of atomising the solution containing EVs. These droplets are rapidly converted into a dry powder using heated gas (Lee, 2002). Spray drying is a continuous process and can be both automated and instrumented for enhanced process control. The reduction in moisture content of particles formed during the spray drying process generally increases the stability of the biopharmaceuticals in these particles: the residual moisture acts as a plasticiser to reduce glass transition temperature of the particle solid state, and its presence may also enhance chemical instability. Critical process parameters such as the rate at which EV solution is being fed into the system, the atomisation pressure and outlet temperature, can all affect the stability of the EVs and their cargo. These critical process parameters must therefore be identified and maintained within a narrow window (Masters, 1972). Behfar (2016) patented a technique to encapsulate the platelet rich solution EVs as a candidate for wound healing (US20160324794A1). However, further investigation is needed to apply this technique more broadly to the manufacturing and storage of EV-based therapeutics.

### Challenges Associated With EVs Preservation

In order for EV-based therapeutics to be manufactured and used reproducibly, storage conditions must have minimal impact on EV structural integrity. The following section will discuss parameters known to affect EV composition, biological potency and structural integrity.

#### Storage Temperature and Shelf Life

There have been a number of studies conducted to determine the most favorable storage conditions for EVs. Focusing on EVs with intended therapeutic applications, EVs from human embryonic kidney (HEK) 293T cells, endothelial colony forming cells (ECFCs) and MSCs report -20°C as the highest temperature in which EVs are stable (Sokolova et al., 2011). These results are in line with the standard preservation temperature reported by ISEV for EVs storage. In contrast, another study has reported that -70°C is the best long-term storage temperature for EVs isolated using the Exo-Quick kit (System Biosciences, Palo Alto, CA, United States) (Lee et al., 2016).

#### Freeze Thaw Stress

While freeze-thaw cycles do not affect the stability of EVs isolated from plasma and exosomal miRNA and different cell types like HEK293T, ECFCs and MSCs (Sokolova et al., 2011; Lv et al., 2013; Ge et al., 2014), other studies show that EVs can be structurally susceptible due to the exposure of vulnerable phosphatidylserine to repeated freeze-thaw cycles (Wu et al., 2015b; Maroto et al., 2017). This is an area that must be deconvoluted as EV-based therapeutics are being developed, in order to establish a clear product stability profile as required by regulatory bodies.

## A Bioengineering Approach to Manufacturing and Enhancing EV Stability

### Overcoming Aggregation in EV Preparations

A preparation of EVs can be considered as a colloid – a solution in which microscopically dispersed particles are suspended (Hood et al., 2014). From this perspective, there are several known phenomena that can be applied to EVs, providing a rationale underlying the basis of possible approaches that can be used to increase the stability and quality of stored EVs. One of the major challenges in EV storage, particle aggregation, occurs when inter-particle attraction is greater than repulsion. Such interactions are governed by factors such as surface charge, hydrophobicity and fluidity (Takeuchi et al., 2000). Strategies to prevent EV aggregation must therefore modify these factors to increase inter-particle repulsion and stabilize the colloidal solution.

**FIGURE 3:**
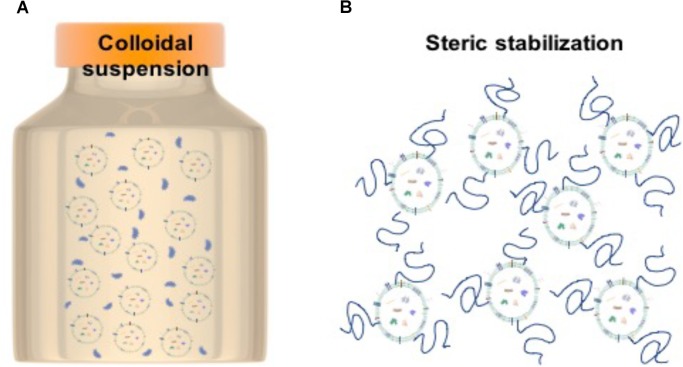
Stabilization strategy for EVs. **(A)** Particles in colloidal suspension. **(B)** Steric stabilization achieved by polymer chains attached to particles to decrease inter-particle interactions.

Although EV biology is a relatively new field, EVs share many overarching structural features with liposomes – lipid bilayered vesicles that have been well-studied due to their utility as drug delivery vehicles. Looking toward liposome studies, the use of hydrophilic polymers as steric stabilizers may be a good strategy for preservation of a colloidal system. When using polymers, it is thought that the hydrophilic chains extend from the liposomes out into the solution thereby stabilizing the system so that the individual particles remain well-dispersed (Figure 3).

The most common polymer used in liposome stabilization is PEG (polyethylene glycol). Advantages of PEG include the fact that it is non-fouling, well-tolerated by the body, can be obtained in a wide range of molecular weights and end-group chemistries and that it is FDA approved for a range of medical applications (Hasan, 2017). There are many examples in which liposomes have been PEGylated, i.e., the PEG chains are incorporated within the lipid bilayer during synthesis (e.g., PEGlyated liposomes to incorporate itraconazole, antifungal agent, as well as dopamine-loaded PEGylated immunoliposomes; Kang et al., 2016; Dzieciuch-Rojek et al., 2017). Although effective, such a strategy is unsuitable for EV stabilization. Coating the particles in polymer can have a similar effect and would be a much more suitable strategy for EV preservation, allowing EVs to be stabilized by the simple addition of polymer to the isolated preparation. Other polymers that have been used include the synthetic polymer PVA (polyvinylalcohol), and the naturally derived polysaccharides OPP (*O*-palmitoylpullulan), chitosan, and hyaluronic acid (Sehgal and Rogers, 1995; Takeuchi et al., 2000; Manconi et al., 2017). Specific stabilization of EVs has thus far been limited to the use of trehalose. Addition of trehalose to solutions of EVs was proven to enhance colloidal stability during electroporation, for the modification of EV cargo (Hood et al., 2014). Addition of 25 mM trehalose to EVs derived from pancreatic beta cells was observed to narrow the particle size distribution (i.e., increase the stability) and improve the particle yield (Bosch et al., 2016), presumably by also reducing loss of EVs through interactions with the walls of the storage vessel.

### Biomaterial Scaffolds for EV Stability and Delivery

The matrix of tissues in the body hosts a population of vesicles, often termed matrix bound vesicles (MBVs) (Shapiro et al., 2015; Huleihel et al., 2016). In a similar manner to the protection of growth factors by sequestration and release from the extracellular matrix (ECM), the binding of these vesicles has a vital role in enhancing their stability and biological availability. Although, there is still debate as to whether MBVs possess all of the characteristics required to be defined as an EV, there is also evidence that EVs can bind to ECM components; for example, a study by Narayanan et al. showed binding of MSC-derived EVs to bind to both fibronectin and collagen type I in the ECM (Narayanan et al., 2016). Such interactions between EVs and the ECM are likely mediated by adhesion receptors, known to be present on the exosomal membrane, including integrins, tetraspanins, and ICAM-1 (Escola et al., 1998; Thery et al., 1999, 2001; Rana and Zoller, 2013).

In the case of MBVs, interaction with the matrix has proven to enhance their stability. MBVs can survive chemical, enzymatic and detergent-based treatments and subsequently induce changes in cellular behavior (Huleihel et al., 2016). These intriguing findings indicate that incorporation of EVs with ECM or biomaterial components may be a powerful tool to both enhance EV stability and provide a controlled spatiotemporal release within the body. This premise is supported by a few early studies in which EVs have been incorporated into biomaterial constructs for delivery. For example, Zhang et al. (2016) stabilized MSC-derived EVs by incorporation into porous tricalcium phosphate (β-TCP) scaffolds. In doing so they demonstrated that EVs could be released over several days and further that the function of these EVs in promoting bone repair was retained. In another study, Shi et al. combined MSC-EVs with a hydrogel synthesized from chitosan and silk, showing that EVs incorporated into the biomaterial could be released over time and retained their function to improve wound healing (Shi et al., 2017). Although, in its infancy, these studies uniting EV biology and bioengineering provide an exciting glimpse into future applications of biomaterials to preserve and deliver EVs for therapeutic application.

## Future Directions

Given that EVs largely retain the properties of their cells of origin, it is unsurprising that cell therapy companies have jumped on this particular bandwagon in order to maximize the proprietary cell lines. For example, Capricor Therapeutics (Beverly Hills, CA, United States) are investigating the clinical potential of CAP-2003, which refer to the EVs produced by their proprietary cardiosphere-derived cells. Capricor has made efforts to evaluate the regenerative potential of these cardiosphere-derived EVs on diseases involving inflammation and fibrosis (Ibrahim et al., 2014). Similarly, cell therapy company specializing in neurological disease, ReNeuron, has sought to do the same with EVs from their proprietary CTX neural cell line, which are currently in Phase IIb clinical trials for US-based patients living with post-stroke disabilities. It is likely that we will need an emergence of EV-based therapeutics from other cell therapy companies as the proverbial penny drops – there is immense value in what was essentially considered a waste product of cell manufacturing.

Regardless of whether EVs will be used for the purposes of regenerative medicine, cancer vaccination, veterinary or agriculture, there is an obvious need to develop methods to reliably store, transport and apply the EVs. Of these considerations, storage of the EVs is perhaps the most critical aspect of the supply chain. The stability of the EVs in their storage medium necessarily dictates the rigidity of the cold chain and will have direct impact on the cost of goods. Investment into technologies that refine the stability of EVs will likely afford significant cost savings downstream. The storage medium will also impact the final formulation of the EV therapeutic as challenges around solubility of injectables and particle size of aerosols must be considered. These factors will have knock-on effects on biodistribution and therapeutic efficacy. As such, rigorous preclinical testing should be designed with this in mind, in order to expedite product development and facilitate regulatory approval.

## Conclusion

The FDA approval of chimeric antigen receptor T cells (CAR-T), Kymriah (Novartis) for refractory B-cell precursor acute lymphoblastic leukemia in August 2017, heralded the dawn of a new age for cell therapies. There are, however, broader implications for these approved cell therapies. Chief amongst these is the growing acceptance of cellular therapies and regenerative medicine in mainstream clinical care. However, the relative high cost of goods remains prohibitive for cellular therapies. Challenges in scalable manufacturing, maintenance of a master cell bank, complex cold chain logistics and ambiguity around product release criteria, have led to lengthy delays in realizing the potential of cellular therapies. While regulatory hurdles for this new class of biologics remain a challenge to be met, it is likely that the relative stability of EVs will see a significantly expedited path to regulatory approval. Furthermore, as critical questions around scalable manufacturing and long-term preservation are answered, EV-based therapeutics may offer a more affordable form of regenerative medicine, thereby increasing market penetration and patient access. In essence, the development of novel preservation protocols tailored for EVs are likely to fast forward the manufacturing process to establish EVs as commercially viable therapeutics.

## Author Contributions

GK, MB, JT, DM, JF, and RL contributed to the writing and editing of this manuscript. GK and JT prepared the figures. GK and MB prepared the tables.

## Conflict of Interest Statement

The authors declare that the research was conducted in the absence of any commercial or financial relationships that could be construed as a potential conflict of interest.

## Footnotes

1 http://www.exocarta.org
